# Partner choice correlates with fine scale kin structuring in the paper wasp *Polistes dominula*

**DOI:** 10.1371/journal.pone.0221701

**Published:** 2019-08-29

**Authors:** Paul John Parsons, Lena Grinsted, Jeremy Field

**Affiliations:** 1 College of Life and Environmental Sciences: Biosciences, University of Exeter, Exeter EX,QD, United Kingdom; 2 School of Biological Sciences, Royal Holloway University of London, Egham, Surrey, TW, United Kingdom; 3 College of Life and Environmental Sciences, University of Exeter, Penryn, Cornwall TR, United Kingdom; Universidade de Sao Paulo Faculdade de Filosofia Ciencias e Letras de Ribeirao Preto, BRAZIL

## Abstract

Cooperation among kin is common in animal societies. Kin groups may form by individuals directly discriminating relatives based on kin recognition cues, or form passively through natal philopatry and limited dispersal. We describe the genetic landscape for a primitively eusocial wasp, *Polistes dominula*, and ask whether individuals choose cooperative partners that are nearby and/or that are genetic relatives. Firstly, we genotyped an entire sub-population of 1361 wasps and found genetic structuring on an extremely fine scale: the probability of finding genetic relatives decreases exponentially within just a few meters of an individual’s nest. At the same time, however, we found a lack of genetic structuring between natural nest aggregations within the population. Secondly, in a separate dataset where ~2000 wasps were genotyped, we show that wasps forced experimentally to make a new nest choice tended to choose new nests near to their original nests, and that these nests tended to contain some full sisters. However, a significant fraction of wasps chose nests that did not contain sisters, despite sisters being present in nearby nests. Although we cannot rule out a role for direct kin recognition or natal nest-mate recognition, our data suggest that kin groups may form via a philopatric rule-of-thumb, whereby wasps simply select groups and nesting sites that are nearby. The result is that most subordinate helpers obtain indirect fitness benefits by breeding cooperatively.

## Introduction

Hamilton's rule states that individuals gain indirect fitness through altruistic or cooperative behaviours that are directed towards genetically related recipients [1]. One way to ensure that help is directed towards relatives is to discriminate kin directly using cues such as pheromones or cuticular hydrocarbons [2,3]. However, true kin recognition is not always possible or may be too costly for evolution to favour it [4–6]. One alternative is to adopt a simple rule-of-thumb of biasing aid towards those in close proximity. This may increase inclusive fitness if the population is structured so that spatially adjacent individuals are of greater than average pairwise relatedness [7]. Structuring in such a way can result from mechanisms such as limited movement or dispersal capability [8], sex-biased dispersal [9,10] and/or natal philopatry [11]. Altruistic and philopatric tendencies may be in a positive feedback loop with increased inclusive fitness benefits due to altruism leading to increased philopatry and vice-versa [12]. This correlation between fine-scale population structure and cooperative tendency has been demonstrated in several species of mammal [13,14], bird [15] and insect [16,17].

While population structuring can provide evolutionary benefits to social species it can also have negative effects. Close proximity of kin leads to increased probability of intraspecific competition [18–20]. Kin competition could even be so strong to completely negate the benefits of kin directed altruism [21], especially in resource limited and/or environmentally unstable environments [22,23]. Genetic structuring can also lead to a higher incidence of inbreeding [24,25], particularly detrimental to haplodiploid insects because it might lead to production of sterile diploid males [26]. These negative effects, alongside other possible abiotic and biotic influences [27], may help to explain why some co-operative species lack kin structuring over physical space (e.g. [28]).

The paper wasp *Polistes dominula* is primitively eusocial, lacking marked morphological castes [29]. The species has expanded its range considerably in recent times, successfully spreading from its origins in the circum-Mediterranean [30] to colder climes in central Europe and the Baltics [31]. It is a notorious invader, establishing in numerous countries far outside its native range, most famously the USA [32]. *Polistes dominula* females overwinter in hibernacula groups comprising 8 to >100 individuals, often originating from more than one natal nest [33,34]. When nest founding starts in spring, foundresses disperse and begin to initiate nests. While nests can be singly founded it is often more common (and more successful) for foundresses to initiate nest formation in small colonies of commonly 5–7 members [29]. Within a colony, foundresses live as cooperative breeders with a single dominant female who lays all or most of the eggs [35]. While there is some evidence that eusocial wasps use facial pattern cues to identify individual nest mates [36], the majority of the literature has focussed on cuticular hydrocarbon profile as a potential cue for discerning kin [3]. It has been suggested that *Polistes* wasps can recognise hydrocarbon profiles of their nest mates but cannot necessarily distinguish relatives from non-relatives that share their natal nest origin or hibernaculum group [37–39]. Nest-mate recognition, as opposed to true kin recognition, is common in social insects [40]. Female *Polistes* are often philopatric, founding new nests in close proximity to their natal nests [34,41].

While previous evidence shows that the mean relatedness among *P*. *dominula* colony members is generally high, colonies may also contain significant numbers of subordinate helpers that are unrelated to the dominant breeder [42,43]. These unrelated subordinates have the potential to gain direct fitness by either sneakily laying eggs in the nest or inheriting the egg-laying position if higher ranked foundresses die [35,43]. In our study population, such direct benefits alone can explain the presence of helpers, although indirect benefits usually represent the larger fitness component if relatedness to the dominant breeder is greater than zero [35].

This study asks two questions (1) Is there micro-scale genetic structuring in populations of paper wasp foundresses? (2) Can any such structuring explain how individuals choose their nesting partners, and which foreign nests they visit? We further discuss whether females are likely to be using a rule of thumb, rather than discriminating kin directly, when choosing nesting partners.

## Methods

### Study organism

At our field sites in a rural part of Southern Spain, near Conil de la Frontera, Cadiz (36°17'10.9"N 6°03'58.1"W), *P*. *dominula* nests are abundant on long, straight hedges of prickly pear cactus (*Opuntia* sp.). At these sites, females from the same generation emerge in early spring after overwintering to found nests either alone or in small colonies. All data used in this study were collected from nests at this founding stage, before any workers had emerged later in the season, when females live as cooperative breeders and no males are present. We were kindly given permission to work on the land by the owners.

### Micro-scale population structuring

We genotyped 1361 wasps from 234 nests also used in the Market Manipulation experiment in [44]. These data represent complete sampling from defined sections of a wasp population meaning that all wasps from all nests present at the time were sampled, apart from a few inaccessible nests. DNA samples were collected prior to the experimental manipulations described in [44,45] and hence represent natural nesting behaviour of the wasps. The 234 nests occurred naturally in three sections of cactus which we refer to as the Backrow, Corner and Island aggregations. These aggregations were separated by a minimum of 40m and a maximum of 220m of bare ground without nesting substrate. The average distance between each nest in Backrow was 43.5cm (SD 64.3), Corner 78.1cm (SD 73.9) and Island 36.4cm (SD 49.9). The aggregations were in turn subdivided into a total of six clusters each separated by stretches of at least 8m containing no wasp nests and where wasps were rarely seen (Table 1). Each cluster contained between 13 and 104 nests (median 29 nests per cluster, Table 1).

**Table 1:** Lengths of cactus nesting substrate at the three nest aggregations and the nest clusters within them, together with the numbers of nests and wasps present.

Aggregation	Island	Corner		Backrow		
Length of Aggregation (m)	16.4	~150		~120		
Cluster	**I1**	**C1**	**C2**	**B1**	**B2**	**B3**
Number of wasps	220	82	69	147	179	664
Number of nests	40	19	13	28	30	104
Length of cluster (m)	16.4	14	10.3	16.4	15.7	47.6

All nests at our field site were tagged and numbered during March 2014. For each nest, we measured its distance along the cactus hedge and its height above ground to the nearest 5cm, and could therefore estimate a 2-dimensional distance between all nest-pairs within each nest cluster. All wasps from all nests within the clusters were collected early in the morning (6:00–7:00) between March 19^th^ and April 25^th^ 2014 and given individual-specific paint marks. Wasps were transported to the laboratory as marking so many in the field was not feasible. At the same time as marking, we obtained a DNA sample by cutting the tarsus from a middle leg [34]. Wasps were then kept at ~4 degrees Celsius until being released close to their nests on the same morning as they were collected, before 11:00.

#### Genotyping

The genotyping protocols are described in [44]. In short, DNA was extracted from tarsus samples and genotyped at nine microsatellite loci used previously in studies of the same population [43–47]. All loci were amplified in a single multiplex reaction using the Qiagen multiplex PCR kit (Qiagen, Venlo, The Netherlands). Microsatellite linkage disequilibrium (LD) and Hardy-Weinberg statistics were assessed and found to be not significant across all 9 markers. See [44] for information on locus heterozygosity and allele frequencies.

#### Analysis: Population structuring between aggregations

To test for genetic structuring at the cactus patch (nest aggregation) level, we calculated pairwise F_ST_ between the 3 aggregations, using GENEPOP on the web [48]. To investigate how the genetic diversity is partitioned across the population we performed analysis of molecular variance (AMOVA). This was set across 3 hierarchical levels—between aggregations, between nests within each aggregation, and between foundresses within nests. This analysis was performed using 999 permutations in GenAlEx 6.52 [49]. As AMOVA requires samples that can be investigated at all 3 levels of analysis, the 22 singly founded nests had to be removed for this particular analysis. We calculated the site level inbreeding coefficient (F_IS_) in COANCESTRY V1.0.1.9 [50].

#### Analysis: Structuring of relatives

Using nest position (height and distance along cactus substrate), geographic matrices of inter- nest distance for the three aggregations and the whole site combined were created in R using the *dist* function [51]. COANCESTRY was then used to calculate the relatedness of every wasp to every other in the population [50]. A small error rate (0.05) was attached to each marker to account for any human error in allele scoring. To minimise any bias from a single measure the Trio Maximum Likelihood option was used to calculate relatedness. The R package *reshape 2* [52] was used to convert the relatedness scores to relatedness matrices, creating one for each aggregation plus a whole site matrix. Spatial autocorrelation analyses were undertaken at the aggregation and the site level in GenAlEx 6.52 using 999 permutations and 1000 bootstraps [49]. This method has been used to assess spatial structure of relatives in other co-operative species [16,17,28]. It is powerful in that it does not assume a simple linear relationship between relatedness and distance, and as such can uncover discrete clusters of related individuals located anywhere in the parameter space. Variable distance classes were used so that the average relatedness among nest mates could be partitioned into one distance class without incorporating individuals from different nests that were in very close proximity. To this end we set the first distance class as 0-9cm, as the smallest distance found between two distinct nests was 10cm.

#### Analysis: Distribution of sisters on a micro-scale

Having established the maximum distance at which individuals remain statistically more likely to be related (up to 15m), we then chose to focus further analysis on the distribution of sisters within this range. In temperate *Polistes*, founding females are normally overwintered females of the same generation, and mothers mate with a single haploid male. The closest possible relationship between two founding females is therefore super-sisters (r = 0.75, i.e. daughters of the same mother and father from the previous year) and the next closest relationship is expected to be cousins (r = 0.1875). Sisters therefore represent the closest possible group of genetic relatives, with a large drop in relatedness to the next-closest relationship. The Full Sib-ship Reconstruction procedure in Kingroup v2 software [53] was used to identify groups of sisters among the nests in each aggregation (primary hypothesis: haplodiploid sisters; null hypothesis: haplodiploid cousins). We asked whether the presence of sisters versus non-sisters was associated with the distance from the nest where each wasp was resident. To do this, we recorded for each individual wasp (N individual wasps = 1361) whether each single other wasp present within 15m in the same nest cluster was a sister or not (across the six clusters, N wasps pairs total = 386,480). We then ran a GLMM [54] with a binomial error structure, with sister versus non-sister as the response variable. Distance between the nest-pairs and cluster ID were included as predictor variables, as we expected clusters to differ due to differences in nest abundance and nest density. Nest ID was included as a random factor, as wasps from the same nest could not be considered independent data points. Significance of predictor variables was assessed by comparing the full model with a reduced model in which the predictor in question had been removed (likelihood ratio test, Chi-Square).

To ask whether wasps tended to belong to the nest in the vicinity that contained the largest number of its sisters, we identified the 10 closest nests within 5m (as very few sisters were found further away than 5m) and counted the sisters that each wasp had on each of those neighbouring nests. We compared this with the number of sisters each wasp had on its own nest. We excluded any wasps that did not have any sisters in its own or neighbouring nests.

### Is partner choice related to population structuring?

During the nest-founding phase in spring, nests newly initiated by single foundresses or groups may subsequently receive additional joiners. As a part of a study published in[45]we recorded the first nest choices of 64 joiners as well as the second nest choices of 25 of these joiners when their first choice nests were removed experimentally. This was done at a different field site, several kilometres apart from the micro-scale data site, across two field seasons in 2013 and 2014. The 64 joiners were each observed joining an established nest, after which we permanently removed the chosen nest (1st nest choice) and its original inhabitants for 25 of them, releasing just the joiner. We then recorded which nest each joiner subsequently chose to join (deemed the 2nd nest choice, see [45] for details).

As described in [45] all nests were tagged and numbered at the field site during March 2013 and March 2014. For each nest, we measured the distance along the cactus hedge, the distance above ground, and the distance into the hedge to the nearest 5cm, and could hence estimate 3-dimensional distances between all nest-pairs. During March-May, we obtained DNA samples from all wasps on both first and second nest choices, as well as from 2–3 randomly selected wasps on each of the remaining nests in the population (~2000 genotyped individuals from ~700 nests, across the two seasons). In summary, the Joiner dataset comprised DNA samples for all of the wasps from the 1st and 2nd nest choices of joiners, as well as 2–3 of the wasps from all other nests at the field site.

#### Female floating behaviour

During the nest censuses carried out as part of the Market Manipulation experiment in [44] (i.e. the Micro-scale Dataset) 60 females were observed at least once sitting on a nest different from their original one. Although we cannot be certain what the wasps were doing on these nests (e.g. just visiting or potentially switching) it is possible that they were prospecting for new colonies. This could involve investigating whether it would pay to switch to a different group, e.g. a group containing more genetic relatives [45]. We will refer to these wasps as “visitors”. We asked whether wasps visited nests that had more sisters than their original nests, by comparing the number of sisters in original versus visited nests using a paired Wilcoxon’s signed rank test.

#### Analysis

We ran simulations to test whether joiners’ 2nd nest choices differed significantly from a random pick in terms of the distance from their 1st nest choices, and in terms of the presence of sisters on 2nd choice nests. For each of these two variables we performed three sets of simulations assuming three maximum dispersal distances. The first set assumed a 10m 3D radius based on the maximum observed distance between 1st and 2nd nest choice of 8.89m. However, most joiners chose a nest much closer than 10m from their 1st nest choice (median distance = 1.21m, mean distance = 1.93m). Therefore, the second set assumed a 5m 3D radius (22 of the 25 joiners chose nests less than 5m away), and the third set assumed a 2.5m 3D radius (representing 20 of the 25 joiners).

For each single simulation we let each of the 20–25 joiners choose a random nest within the defined radius, and then pooled the 20–25 simulated nest choices and calculated the average distance from their original nests to their new nests and the proportion of those new nests that contained sisters. We ran these simulations 1000 times and compared the observed values for distance and proportion of sisters with the distribution of simulated values. P-values were obtained by calculating the proportion of simulated values that were more extreme (higher proportions of sisters and shorter distances) than the observed values and multiplying this proportion by two.

DNA was sampled differently for focal and non-focal nests: all individuals in focal 1st and 2nd choice nests were genotyped (median number of nest residents per focal nest genotyped = 4) while only 2–3 individuals in non-focal nests were genotyped (median number of nest residents genotyped per non-focal nest genotyped = 3). Because of this discrepancy, there was a risk of false negatives in presence of sisters in surrounding nests, deflating the simulated proportion of neighbouring nests with sisters. To account for this, we ran two additional, more conservative, sets of simulations to obtain two alternative observed proportions of nests with sisters, simulating a sampling of first one and then two fewer nest residents in focal nests. In the first set, we randomly chose one resident from each of the focal nests and removed it in each simulation, and then calculated the proportion of nests with sisters. We performed 1000 simulations and then calculated the average proportion. In the second set of simulations we instead removed two randomly chosen residents from each nest. Hence, we compared three different observed proportions of chosen nests that contained sisters with the simulated values from random nest choices: the actual observed proportion (66.7% of nests had sisters), a simulated average proportion with one resident removed per nest (66.1%), and a simulated average proportion with two residents removed per nest (56.7%). In all three sets of simulations (10m radius, N = 21; 5m radius, N = 19; 2.5m radius, N = 17) the maximum simulated proportion of chosen nests that contained sisters (maximum values: 23.8%, 31.6%, and 35.3% respectively; mean values: 3.1%, 7.1%, and 13.1% respectively) were all lower than the three observed values.

## Results

### Micro-scale population structuring

Pairwise F_ST_ was very low between the 3 aggregations, suggesting minimal population structuring at this level (BR vs C = 0.02768, BR vs. I = 0.0131, C vs I = 0.02922). This was corroborated by the AMOVA analysis. Aggregation explained only 2% of the total variance across the data set. Considerably more of the variance was explained among-nests within aggregations at 23%, with the remaining 75% explained within-nests. The inbreeding coefficient (F_IS_) calculated across the population was 0.083.

#### Structuring of relatives

**Fig 1:**
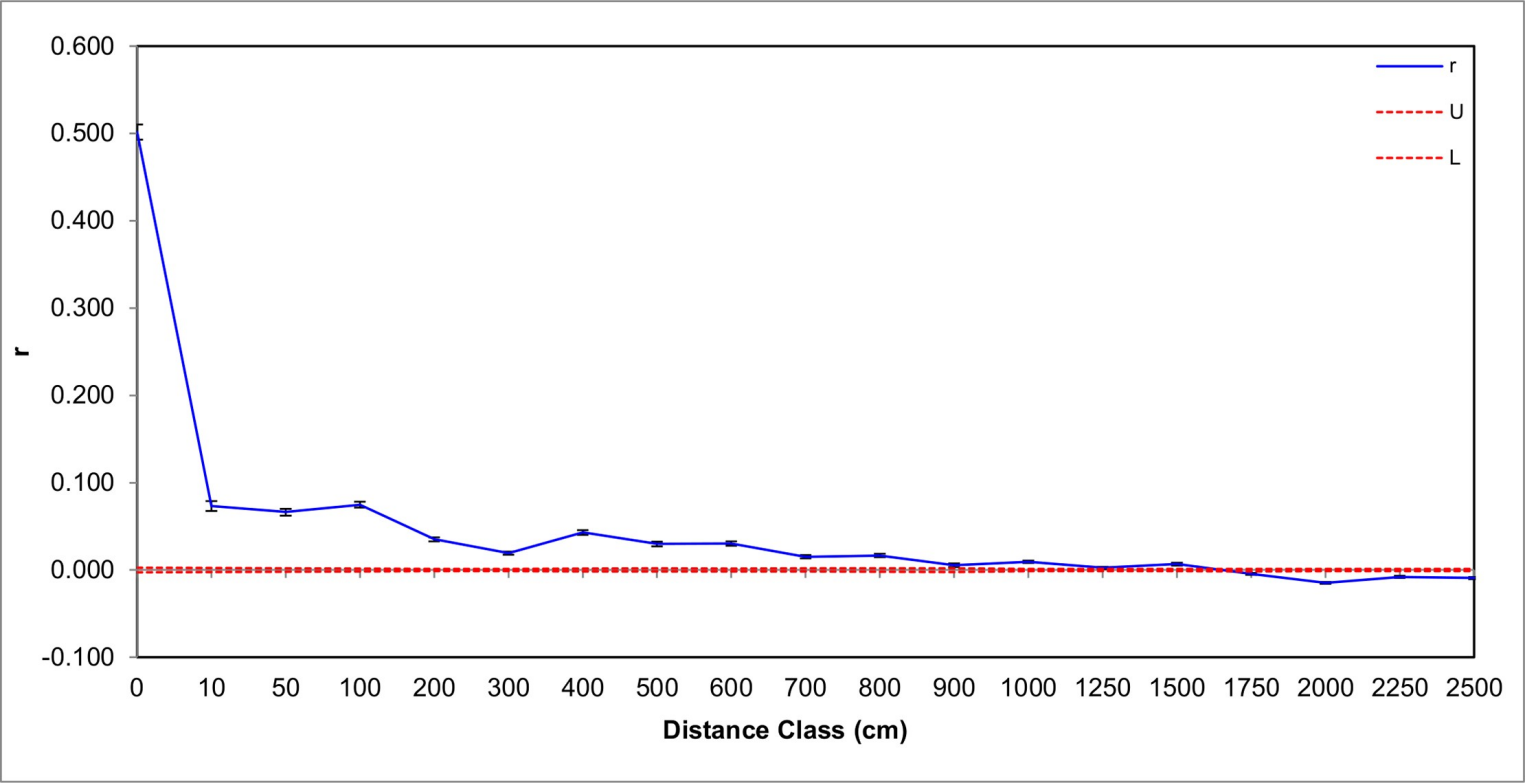
Correlogram of relatedness between pairs of foundresses in relation to the distances separating them, combining all three aggregations from the Micro-scale Dataset. The blue continuous line is the autocorrelation coefficient (r), with black error bars indicating the 95% bootstrapped confidence intervals. The dotted lines represent the upper (U) and lower (L) 95% confidence intervals for the null hypothesis of no spatial genetic structure, generated from 999 permutations.

Spatial autocorrelation analysis performed between every individual in the site level matrix revealed a significant positive relatedness structure up to 15m away from the focal individual (Fig 1). The same result was found using separate matrices for each of the three aggregations, but the maximum distances were lower than for the combined dataset (Backrow– 600cm, Corner– 200cm, Island– 100cm) (S1A, S1B, and S1C Fig)

#### Distribution of sisters on a micro-scale

**Fig 2:**
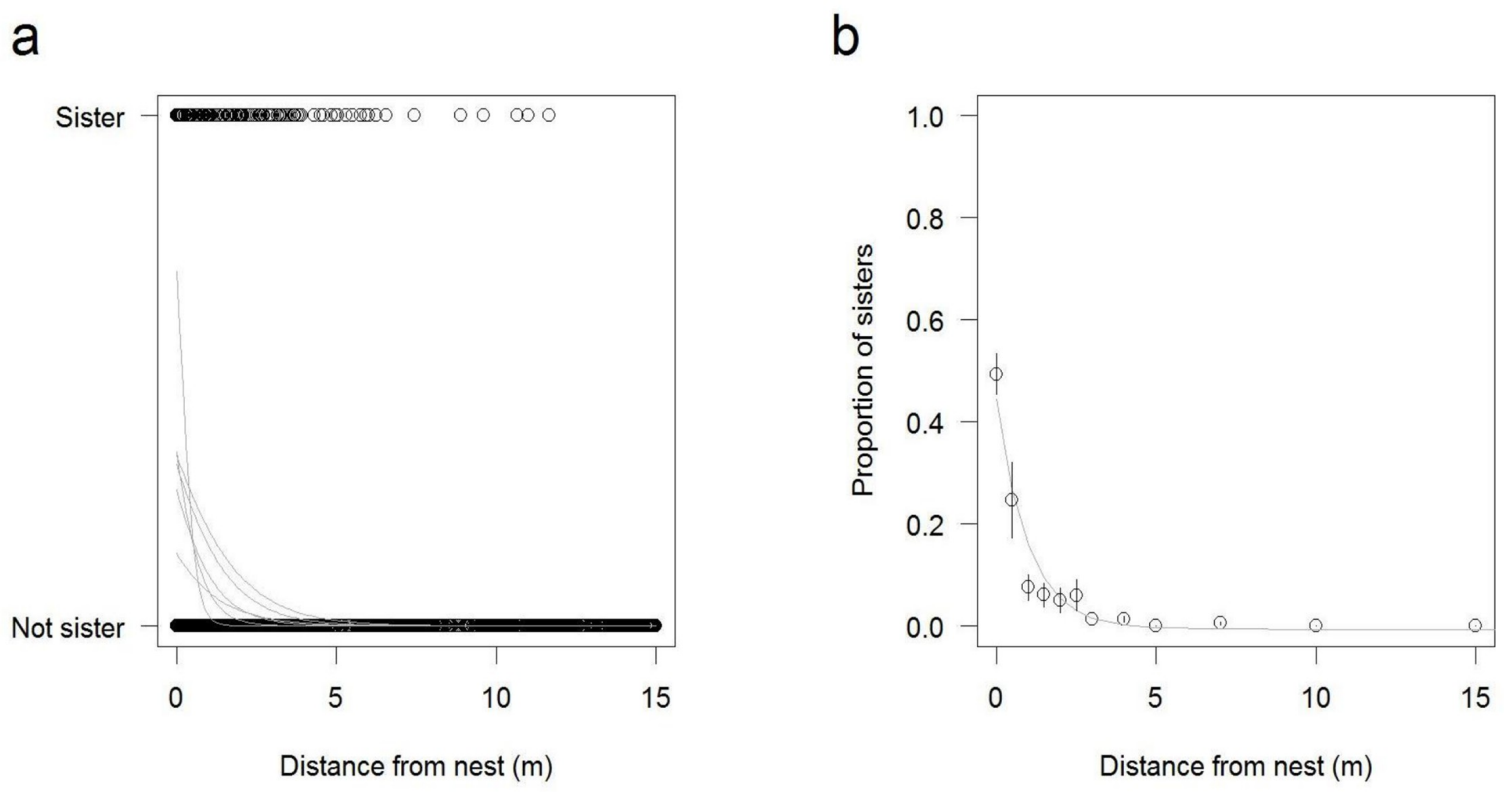
Presence of sisters within 15m of focal nests in the Micro-scale Dataset. a) For each of the 1361 focal wasps, all sisters and non-sisters within 15m are plotted against distance from the nest. Focal nest-mates are at distance = 0. The grey lines indicate the variation between clusters: They show the predicted values from six binomial GLMs each performed on data from one of the six nest clusters, with distance as the only predictor. b) The proportion of sisters found at different distances from focal nests, averaged across the six nest clusters. Error bars are standard errors depicting the variation across the six clusters. The grey line shows the predicted values from an exponential LM on proportional data and does not represent the full statistical model as reported in the Results, which is performed on binary data.

Distance strongly predicted the presence of sisters (Chi-Square = 30075; p < 0.001, Fig 2A). The likelihood that a wasp from a nearby nest was a sister dropped exponentially within the first few meters (Fig 2B). Indeed, the presence of sisters dropped to almost zero after 5m. As expected, the relationship differed significantly between clusters, probably because of differences in nest density (Chi-Square = 17.6; p = 0.0035).

**Fig 3:**
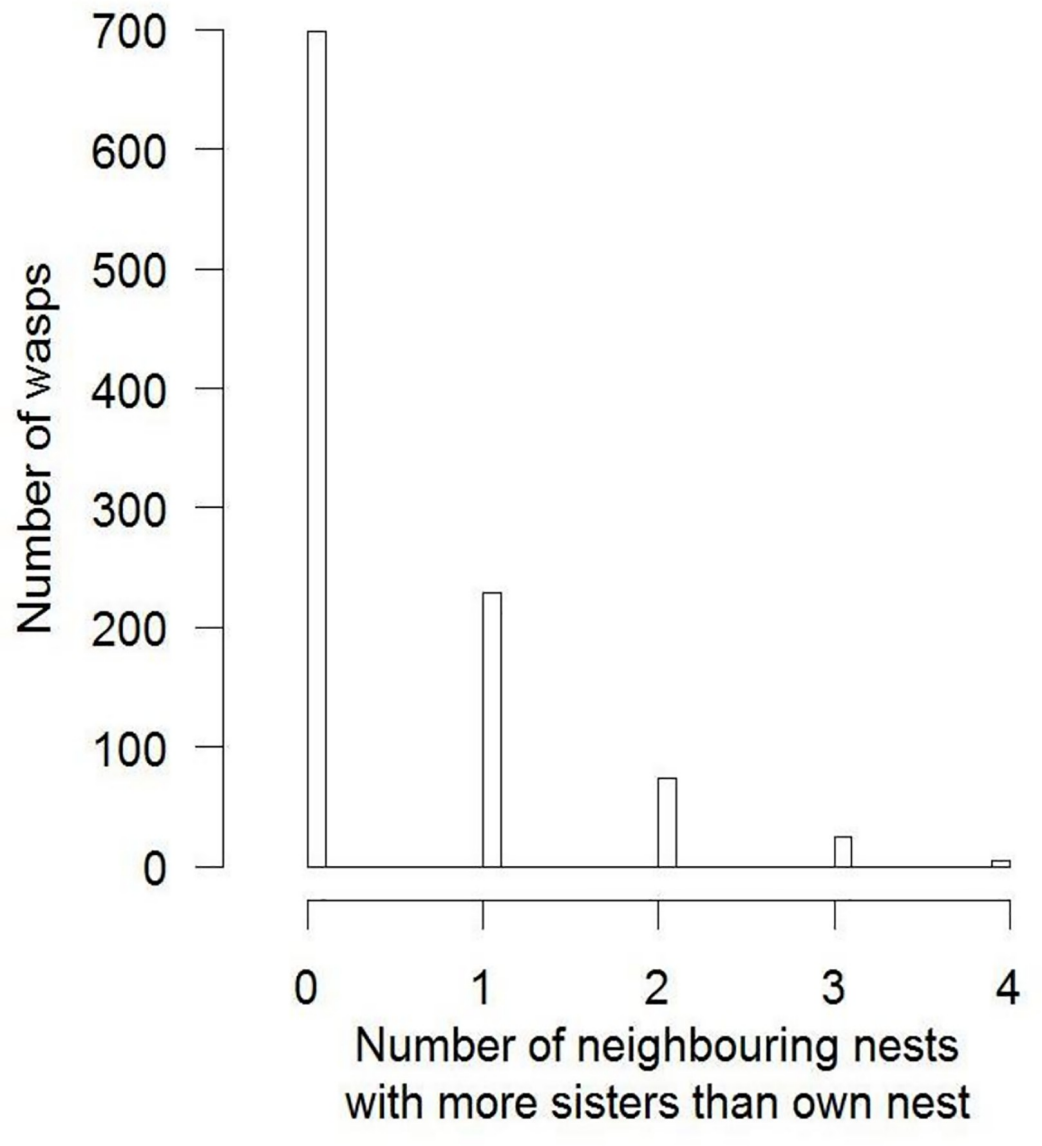
Frequency distribution depicting the number of focal wasps that had more sisters present in each of up to four other nests within 5m than was present in its own nest using the Micro-scale Dataset.

**Fig 4:**
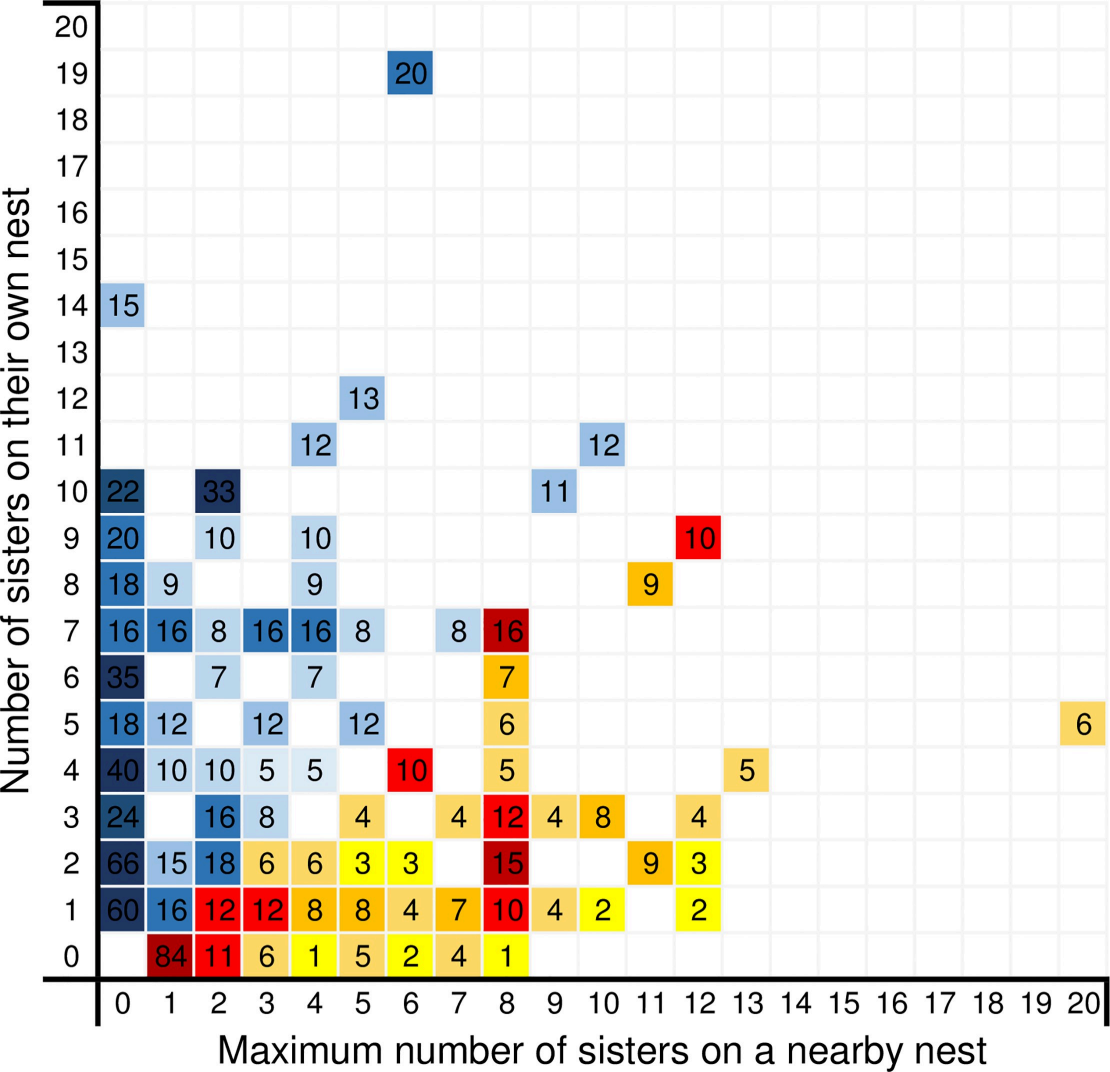
Heatmap depicting the relationship between the number of sisters present on a wasps own nest against the nest within 5m with the highest number of sisters. The heat of a cell and the number within it indicate the wasp count. Cells with a blue hue are wasps residing in a nest that has the largest number of sisters available, Reds and yellows show wasps that are residing in a nest with fewer sisters than one or more other nests.

Of wasps that had sisters present on their own nests or on other nests within 5m, about 2/3 (67.7%; 698 out of 1031 wasps) resided on the nest that had the largest number of sisters (Fig 3). Around half of which (334) reside on the only nests within the sample set that contained sisters (Fig 4). Each of the remaining 32.3% of wasps had fewer sisters in its own colony than in 1 to 4 other colonies within 5m (Fig 3), with 114 wasps residing on nests with no sisters present even though sisters were present on other nests (Fig 4).

### Is partner choice related to population structuring?

When making their first nest choices, almost 40% of joiners chose nests without any sisters in them: 27/64 of joiners chose nests without any sisters even though at least 16/27 had sisters in up to 5 other nests elsewhere in the population. The remaining 37/64 joiners chose nests with at least one sister and 19 out of these 37 chose nests that consisted only of sisters.

With regards to second nest choices, 14/21 joiners had at least one sister in their new nest while the remaining 7 did not have any sisters in their new nest, even though 6 out of these 7 had sisters in up to 5 other nests elsewhere in the population. 12/18 joiners that joined already-established nests (as opposed to initiating new nests) chose a nest that was within the 10 closest nests. Of these, 5 chose the very closest nest. Of the 6 that chose a nest further away than the 10 closest, 3 might have moved far to find a nest with sisters: they did not have sisters in their 10 closest nests but did have sisters in their new, chosen nest.

**Fig 5:**
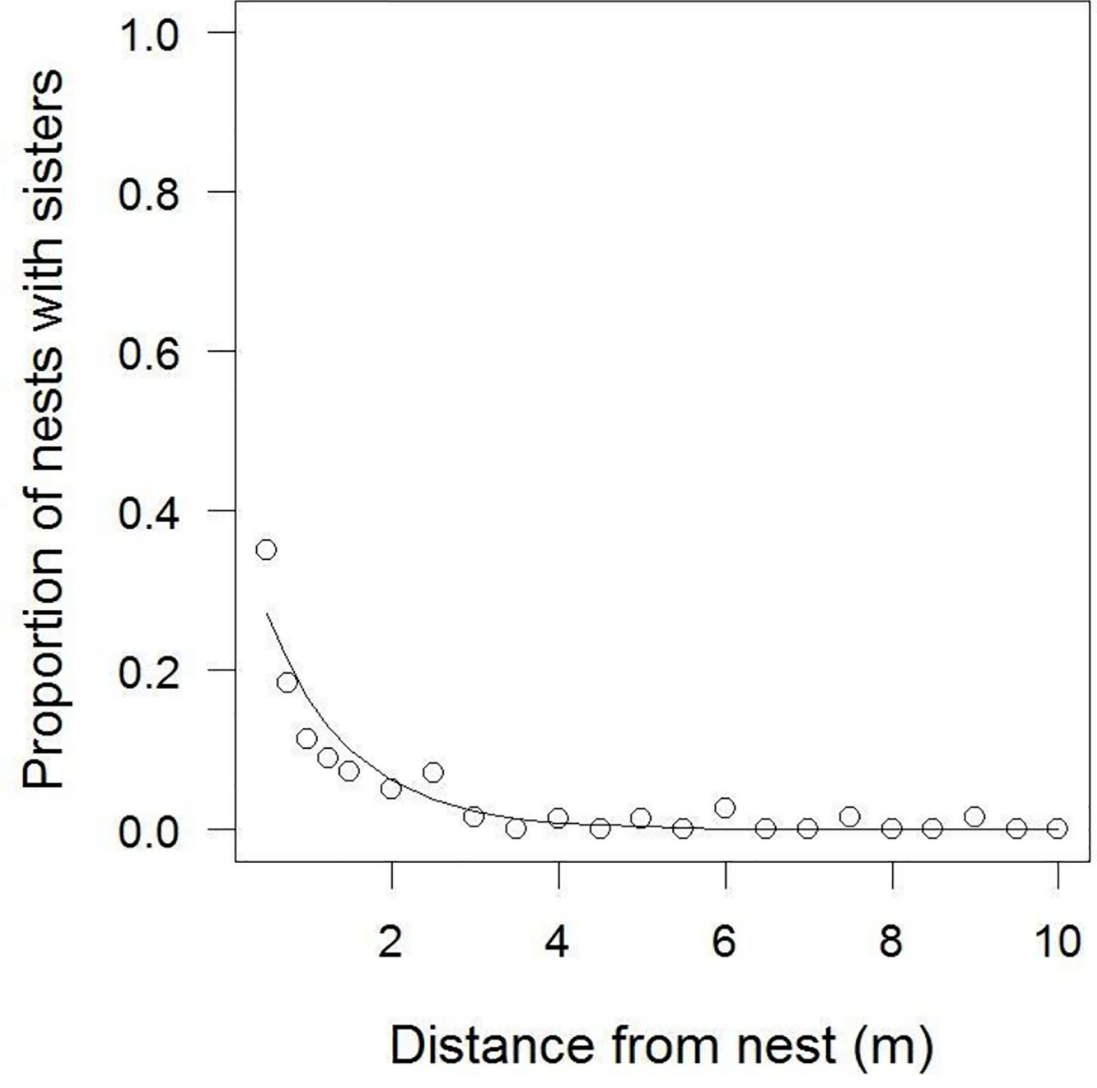
Proportion of nests that had sisters in relation to 3D distance from a focal joiner’s first choice nest, calculated by pooling data for 25 joiners from the Joiner Dataset.

The likelihood that any neighbouring nest contained sisters of a joiner decreased exponentially and significantly with 3D distance from the joiner’s 1^st^ nest choice (F = 177.8, r2 = 0.89, p < 0.001, Fig 5). Correspondingly, joiners chose new nests that were significantly closer in 3D space to their original nests than if they had chosen a nest randomly (simulations assuming 10m and 5m radius: p < 0.001; 2.5m radius p = 0.004). Joiners were also significantly more likely to choose a new nest with sisters present than if they had chosen a nest randomly within 10m, 5m or 2.5m of their 1^st^ nest choices (p < 0.001).

#### Female floating behaviour

The nests that wasps visited were mainly located within 2m of their own nests (median = 0.82m; mean = 1.18m; maximum = 6.02m). There was considerable variation in the number of sisters present on visited versus original nests (from 7 more to 14 fewer sisters in the visited nests, median = 0). Wasps did not consistently visit nests that had more sisters than their own nest (Paired Wilcoxon signed rank test with continuity correction, V = 321.5; p = 0.29; N = 60).

## Discussion

We document an extremely fine spatial structuring of relatives within a paper wasp population, despite finding no genetic structuring between three naturally occurring large aggregations within the field site. Indeed, from the perspective of a single wasp, the chance of locating relatives beyond 15m from its nest is very low, with full-sibling encounters almost non-existent beyond just a 5m radius. Furthermore, we show that wasps that visit and permanently join new nests tend to do so within this 5m radius, maximising their chances of joining a group that contains full sisters.

We found that joiners chose new nests that were significantly closer in 3D space to their original nests than if they had chosen a nest randomly within their cluster, and that those nests were more likely to have sisters present than a random nest. While we cannot disentangle these two correlated factors, i.e. a preference for nests with sisters versus a preference for nests nearby, one possibility is that wasps do not or cannot actively seek out sisters as cooperative partners. First, we found that almost a third of all wasps (333/1031) resided in groups that did not contain the largest number of sisters available among the surrounding nests. Second, 25% (16/64) of joiners chose nests with no sisters in them at all, despite having sisters in nearby nests. When it came to making their second nest choices, again 29% (6/21) chose nests with no sisters in them despite having sisters in nearby nests. Third, when wasps visited other nests, these nests did not contain more sisters, indeed they ranged from containing 7 more to 14 fewer sisters than their original nests. Another way of viewing these findings, however, is that they simply represent imperfect recognition of kin or nest-mates, and/or the pursuit by some individuals of direct fitness strategies for which associating with kin would be disadvantageous. For example, approximately 30% of joiners end up as dominant breeders in the groups that they join [45]. One indication we have that wasps might in some cases actively seek out sisters is that three joiners with no sisters within the ten closest nests chose new nests further away that did indeed contain sisters. However, it is possible that these three wasps chose nests close to their (unknown) natal nests, rather than specifically sought out sisters.

While acknowledging the possibility that direct recognition may contribute to group formation, we focus the remaining discussion on other mechanisms. If joiners cannot use direct kin recognition cues to choose new nests, they may instead use a rule of thumb–‘join nests within only a few metres of natal nest/overwintering site’ in order to maximise chances of cooperating with kin. Indeed, when we forced joiners to make a second nest choice, 28% (5/18) chose the very nearest nest and another 39% (7/18) chose another one of the closest ten nests which were located within just a few metres. Although it is potentially less reliable than directly identifying kin, such a rule would enable foundresses to relatively inexpensively increase the probability of co-operating with relatives and obtaining indirect fitness benefits in a structured population. This association by proximity rule cannot be ruled out as an explanation for kin co-operation in other species [55,56]. In fact, it seems likely that simple rules of thumb often dictate key behaviours that would naively be assumed to rely on recognition cues (e.g. prey identifying the level of threat posed by different potential predator species [57] or hosts discriminating the eggs of intraspecific brood parasites [58]).

Our limited support for true kin recognition is consistent with current data in the literature [59]. It seems possible that *P*. *dominula* foundresses can recognise their natal nest material and potentially individuals from their natal nests or overwintering site using cuticular hydrocarbon cues [37,60]. However, due to potential sharing of odours between individuals, the utility of these as a strict method of kin recognition is debatable [40,61]. For example, it is likely that their use as a kinship cue is confounded by the mixing of scents whilst overwintering [39]. While overwintering aggregations do generally contain individuals of high relatedness, they can also contain many unrelated individuals [42]. It is possible that associations between overwintering individuals are a product of proximity rather than kin sorting, mirroring our predictions from joining behaviour. Relatedness in these overwintering associations is on average slightly lower than relatedness in early nest associations [42], tentatively suggesting some selection of more closely related kin on nest foundation. However, this could be achieved by individuals returning to spots near to their natal nests after overwintering, rather than via direct kin recognition. Although the locations of natal nests and hibernacula are unknown for our wasps, previous studies have shown directly that *Polistes* are often highly philopatric [41,62].

Spatial structuring of kin is not universal [63–65], with increased competition between relatives [18,19] and/or increased probability of inbreeding depression [24,25] often cited as drivers of dispersal. The kin structuring found here suggests that these potential drivers of dispersal are out-matched by kin-selected benefits of philopatry (and other potentially unmeasured factors). Kin competition could take several forms in *P*. *dominula*. First, usurpation of nests by kin would diminish inclusive fitness benefits gained through philopatry. Although usurpation rate in *P*. *dominula* is lower than that of many other *Polistes* species [66,67] it is not insignificant (1–2% successfully usurped per day) [68,69]. It is not currently known how closely related usurping foundresses are to the individuals present on the nests they are attacking or the distances from their natal locations they attack. A potential interesting extension to the rule of thumb that could be investigated is “join nests within a few meters, usurp beyond this”. A second form of competition among kin that could select against philopatry is for resources [20]. This, however, is likely to be a weak force in our study population; foraging effort is not correlated with local nest density [44,45]. An increased probability of inbreeding can select against kin structuring, especially in haplodiploid species where it can lead to the generation of sterile diploid males [26]. The inbreeding coefficient calculated in our study (0.083) is slightly higher than that recorded previously for the species (0.04 - [70], 0.01 - [71]) but is much lower than found in several other *Polistes* species (up to 0.52 in *P*. *exclamans*) [72,73]. Our slightly positive value suggests some level of inbreeding within our population but this is unlikely to be sufficient to cause limiting depression. We can only speculate on how genetic diversity is maintained despite the philopatric tendency of females. Male biased dispersal that yields a regular influx of breeding males from distant populations is one possibility [9]. One way in which kin do compete directly is for inheritance of the dominant, egg-laying position in multi-female groups. By dispersing, a foundress could instead compete with non-kin, and unrelated foundresses have been shown to work no harder and occupy no lower ranks in the inheritance queue than relatives [43]. As well as the possibility of obtaining indirect fitness benefits through cooperating with kin, it could be that predation pressure is a driver of natal philopatry [74]. If the chance of predation away from nests is high [75], it could pay foundresses to minimize dispersal by overwintering close to their natal nests and by founding new nests close by the following spring. This might lead to overwintering aggregations comprising mainly relatives, with wasps joining the first aggregation that they happen upon. On emergence from hibernation in spring, wasps might similarly do best to found nests as soon as a viable location is found.

Although we have found clear evidence of fine-scale kin structuring among founding females, this does not translate to significant structuring at the between-aggregation level in our study population. Genetic structuring between close aggregations has been suggested in some other eusocial insects [16], but is rare in *Polistes* species [72,76]. Indeed, mirroring our results, Lengronne *et al*. [70] found little evidence of structuring among three aggregations of *P*. *dominula* at our sampling location of Conil de la frontera on a similar spatial scale, albeit with a far smaller sample size than ours. This result is not particularly surprising. An individual *Polistes* foundress can produce large numbers of reproductives, and as such relatively few successful dispersers per generation could have a swamping effect on population structuring. Male-biased dispersal that may function to reduce inbreeding depression will also maintain gene flow between aggregations as a by-product [9].

In conclusion, we report fine scale population structuring among females in a primitively eusocial wasp, despite no genetic structuring between larger aggregations. The chance for an individual to find relatives decreases exponentially as a function of distance, and full sisters are almost impossible to find 5m away from a focal nest. Although we cannot rule out a role for direct recognition, this structuring suggests that philopatry rather than direct kin recognition, might be the main process driving the formation of groups of relatives in *P*. *dominula*. This is consistent with the fact that joiners often choose nests without any close relatives, despite having sisters on other nests nearby. A simple rule of thumb, ‘settle close to your origin ‘, would then be what ensures that indirect fitness benefits can be obtained by most subordinate helpers.

## Supporting information

S1 Fig **Correlograms of relatedness between pairs of foundresses assessed at variable distance classes for the 3 aggregations separately a) Backrow, b) Corner, c) Island**. Thick continuous line is the autocorrelation coefficient (r). The red dotted line dictates the upper and lower 95% confidence on a null hypothesis of no spatial genetic structure generated from 999 permutations. The black error bars show the 95% bootstrapped confidence intervals for r.
(DOCX)

S1 File Nest location and foundresses genotype dataset. Nests are split by aggregation. Nest location is given as a distance along the cactus hedgerow in cm (length) and the height up the cactus. Second sheet lists the wasp genotypes and inhabiting nest.
(XLSX)
