# Supplementary material for: Partner choice correlates with fine scale kin structuring in the paper wasp Polistes dominula
Source: PLoS One. 2019 Aug 29;14(8):e0221701. doi: 10.1371/journal.pone.0221701 (PMC6715180; doi:10.1371/journal.pone.0221701)
Supplement: S1 Fig — Correlograms of relatedness between pairs of foundresses assessed at variable distance classes for the 3 aggregations separately a) Backrow, b) Corner, c) Island. Thick continuous line is the autocorrelation coefficient (r). The red dotted line dictates the upper and lower 95% confidence on a null hypothesis of no spatial genetic structure generated from 999 permutations. The black error bars show the 95% bootstrapped confidence intervals for r. (DOCX) [file pone.0221701.s001.docx]

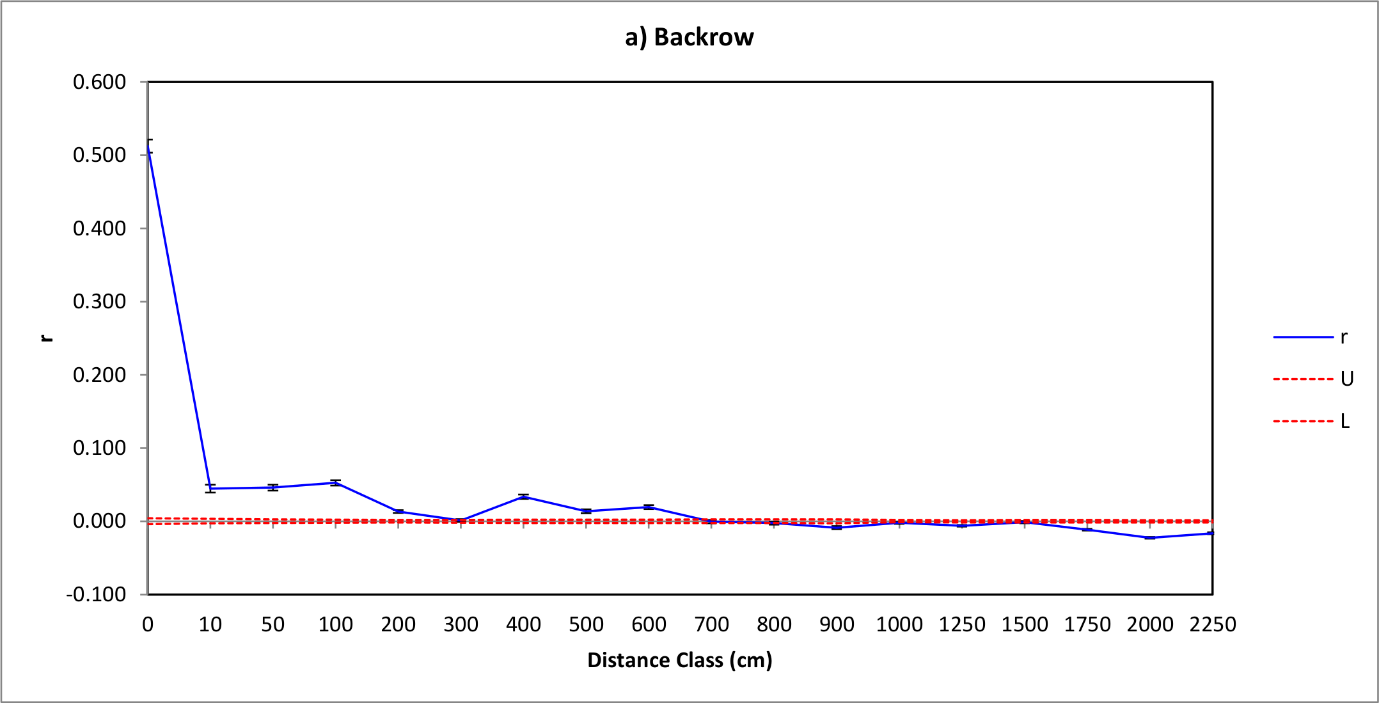


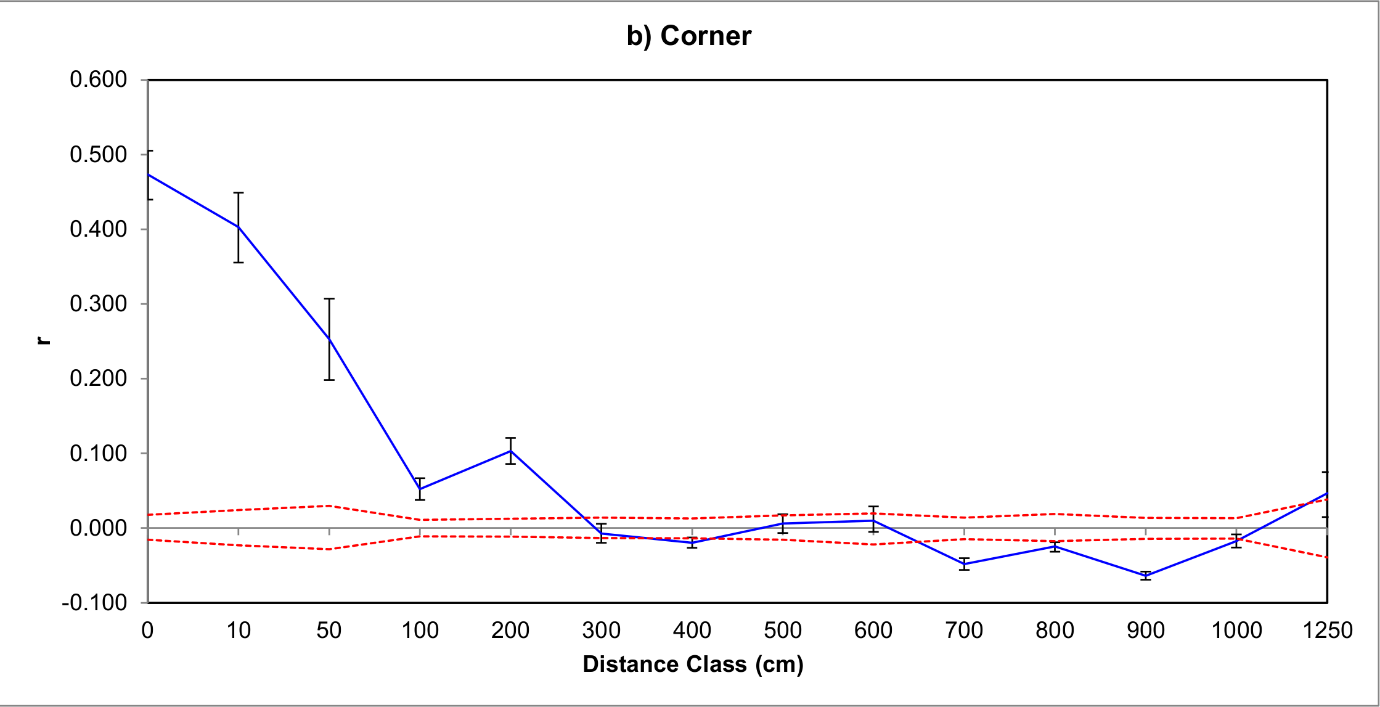


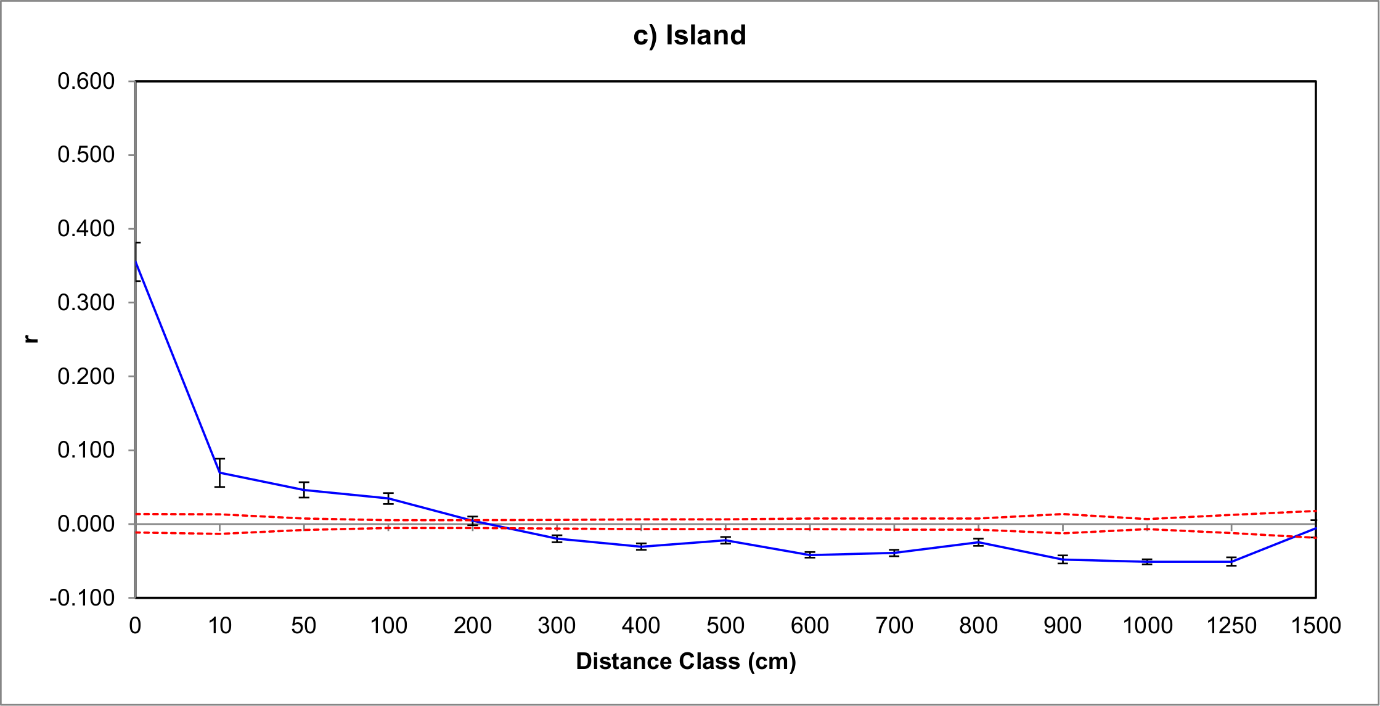


**Fig. S1 Correlograms of relatedness between pairs of foundresses assessed at variable distances classes for the 3 aggregations separately a) Backrow, b) Corner, c) Island**

Thick continuous line is the autocorrelation coefficient (r). The red dotted line dictates the upper and lower 95% confidence on a null hypothesis of no spatial genetic structure generated from 999 permutations. The black error bars show the 95% bootstrapped confidence intervals for r.
